# The Effectiveness of Personalized Robot-Assisted Rehabilitation on Fall Risk: A Retrospective Controlled Study with a 6-Month Follow-Up

**DOI:** 10.3390/s26134088

**Published:** 2026-06-27

**Authors:** Letizia Castelli, Anna Maria Malizia, Alessandra Pedico, Sofia Tarquini, Claudia Loreti, Lorenzo Biscotti, Silvia Giovannini

**Affiliations:** 1 Department of Neurosciences, Università Cattolica del Sacro Cuore, 00168 Rome, Italy; 2 UOSD Riabilitazione Multidimensionale e Tecnologie Integrate, Fondazione Policlinico Universitario A. Gemelli IRCCS, 00168 Rome, Italy; 3 Department of Emergency, Anaesthesiology and Intensive Care Medicine, Fondazione Policlinico Universitario A. Gemelli IRCCS, 00168 Rome, Italy; 4 Unità Supporto Amministrativo Dipartimenti Universitari, Università Cattolica del Sacro Cuore, 00168 Rome, Italy; 5 Department of Geriatrics and Orthopaedics, Università Cattolica del Sacro Cuore, 00168 Rome, Italy

**Keywords:** risk of falls, balance, robotic rehabilitation, hunova

## Abstract

Falls represent a severe public health issue and a primary cause of impairment across both geriatric and adults clinical populations, underscoring the importance of early and tailored prevention. Research evidence shows that technology-assisted rehabilitation may enhance balance and reduce fall risk; however, long-term real-world evidence remains limited. This retrospective study evaluated the impact of personalized balance training by hunova^®^ robotic platform on fall risk in a heterogeneous clinical cohort (n = 355; mean age 58.24 ± 19.63 years) comprising neurological and orthopedic conditions. Fall risk was assessed using the Silver Index, a sensor-based robotic posturographic score to predict 12-month fall probability at baseline (T0), after 6 weeks (T1), and at 6 months (T6). Robotic rehabilitation was provided to 162 patients (TREAT-G), while 193 served as a control group (NOTREAT-G). TREAT-G demonstrated a significant reduction in fall risk over time (*p* = 0.036), with improvements from T0–T1 (*p* < 0.001) and T0–T6 (*p* = 0.009). Domain-specific analysis showed significant increases in limits of stability, sit-to-stand, and gait speed (*p* < 0.001). Significant time × group interactions were observed for limits of stability (*p* = 0.004) and sit-to-stand (*p* < 0.001). Based on the occurrence of falls in the previous six months, the sample was then divided into ‘Fallers’ (at least one fall) and ‘No-Fallers’ (no falls). Subgroup analysis revealed significant improvements in ‘Fallers’ (*p* = 0.016) and ‘No-Fallers’ (*p* = 0.034) following treatment in contrast to untreated patients—both ‘Fallers’ and ‘No-Fallers’. These results indicate that tailored robot-assisted therapy with hunova^®^ significantly reduces risk of fall and improves long-term dynamic balance. This study supports the clinical integration of robotic assessment and therapy as a robust tool for proactive fall prevention in diverse clinical settings.

## 1. Introduction

Fall is defined as “a sudden, unintentional, unexpected downward movement from a standing, sitting or lying position” [1]. According to the World Health Organisation (WHO), falls are the second leading cause of death from unintentional injuries globally after deaths caused by road traffic accidents, with approximately 684,000 deaths per year. Notably, an estimated 80% occur in low- and middle-income settings. Beyond fatal outcomes, the WHO estimates that approximately 37 million people affected by falls require medical treatment each year [1] and that falls are responsible for increased disability and healthcare expenditure as well as a reduction in remaining independence [2,3].

A complex interplay of intrinsic and extrinsic risk factors significantly amplifies an individual’s susceptibility to falls. Intrinsic risk factors include age and gender [4], health status [5], mobility and sensory impairments [4], and cognitive abilities [6]. Extrinsic risk factors include environmental hazards, footwear, and assistive devices [4,5].

While falls are traditionally recognized as a critical indicator of vulnerability in geriatric populations—affecting approximately 40% of community-dwelling adults over the age of 65 annually [7,8]—postural instability and fall risk also heavily burden younger adult populations suffering from acute or chronic neurological, musculoskeletal, or post-traumatic disorders [9]. The post-acute discharge period represents a phase of heightened vulnerability across these cohorts, necessitating the development of robust, continuous screening and targeted prevention programs [10]. Current clinical guidelines recommend adopting multidisciplinary, multifactorial approaches to mitigate modifiable risk factors [2]. To reduce the incidence and consequences of falls as well as the healthcare costs associated with managing them, the WHO and its “Falls Prevention Network” emphasise the need to implement structured management and prevention programmes, backed by adequate financial resources and firmly integrated into geriatric care pathways [2]. Exercise is still the most reliable single-component technique for lowering fall risk, especially when balance and functional mobility are the focus [11]. Multifaceted programs that include exercise, assistive technology, and fall risk assessments provide further advantages [12,13,14]. Technology-enhanced exercise programs, such as sensor-based feedback, exergames, tele-rehabilitation, and robotic systems, have arisen to overcome these obstacles by facilitating objective evaluation, adaptive training, and scalable delivery. Robotic-assisted balance and gait training, such as on a treadmill, offers high-repetition, task-specific practice with standardized progression and real-time feedback, potentially improving training intensity, motor learning, and safety [15,16].

Hunova^®^ (Movendo Technology Srl, Genoa, Italy) [17,18,19] is a sophisticated robotic platform designed for the evaluation and rehabilitation of postural control, featuring sensorized electromechanical surfaces that facilitate the quantitative analysis of stabilometric parameters and the provision of adaptive training in both static and dynamic environments. It facilitates an objective and very sensitive evaluation of balance in contrast to conventional clinical assessments, demonstrating strong concordance with established gold standard devices like EquiTest^®^ [20].

In clinical practice, hunova^®^ is utilized in neurorehabilitation (e.g., stroke, Multiple Sclerosis, Parkinson’s disease), providing personalized protocols based on multimodal feedback that enhance motor learning and neuroplasticity [21]. Randomized controlled experiments have shown that the incorporation of hunova^®^ training with traditional rehabilitation markedly enhances both static and dynamic balance as well as functional independence in post-stroke patients. These enhancements correlate with decreased postural sway and augmented trunk control, factors directly linked to fall risk [22,23]. Ultimately, the use of hunova^®^ signifies a novel promising approach to enhancing balance and mitigating falls in high-risk clinical groups.

Despite these promising technological advancements, existing evidence is limited: most clinical trials are characterized by small sample sizes, highly selected populations, and short-term evaluations immediately following interventions. Therefore, large-scale, real-world studies with longitudinal follow-up are needed to determine whether robotic rehabilitation can produce sustained improvements in objectively measured fall risk and, ultimately, contribute to reducing fall incidence in heterogeneous clinical populations. In this study, ‘fall risk’ is defined as the probability of experiencing at least one fall during the 12-month period following the initial assessment. This definition aligns with the validated methodology established by Cella et al. [24], which utilizes the Silver Index algorithm on the Hunova robotic platform to prospectively monitor and categorize individuals as fallers or non-fallers.

To address this gap, the primary objective of this retrospective controlled study was to analyse quantitative parameters obtained from a large, heterogeneous clinical cohort of adult patients who underwent robotic balance assessments and personalized training via hunova^®^ robotic platform. Specifically, we examined the effects of personalized robotic treatment on improving balance and reducing the risk of falls analysing these effects over time.

## 2. Materials and Methods

This is a longitudinal retrospective observational study. All data included in the study were collected as part of the outpatient fall prevention program active at our hospital, and these are data that are routinely collected. The study included all outpatients of the Multidimensional Rehabilitation and Integrated Technologies Operative Unit of the Fondazione Policlinico Universitario “A. Gemelli” IRCCS who, between January 2022 and December 2025, met the following inclusion criteria: (i) aged between 20 and 95 years and (ii) having undergone at least one fall risk assessment using the hunova^®^ robotic platform. The study protocol was conducted in accordance with the Declaration of Helsinki and was approved by the local Institutional Ethics Committee, which granted a waiver of informed consent due to the retrospective nature of the pseudonymized data extraction.

Data from all patients included in the study were collected at the following timepoints: first visit (baseline, T0), second visit (6 weeks, T1), and third visit (6 months, T6). Specifically, at T0, demographic and anthropometric data were collected, along with clinical characteristics: age, sex, educational level, body mass index, date of examination, medical history, current medical conditions, ongoing pharmacological treatments, date of the first visit, number of falls in the last 6 months and the Silver Index value recorded using the hunova^®^ robotic platform; at T1, the Silver Index value recorded using the hunova^®^ robotic platform was collected, together with information regarding whether any technological balance treatment sessions had taken place and the number of such sessions using the hunova^®^ robotic platform; and at T6, the number of falls in the last 6 months and the Silver Index value recorded using the hunova^®^ robotic platform were collected.

The primary outcome of interest was the Silver Index, a predictive algorithm designed to quantify an individual’s overall risk of falling [19,24,25,26,27] using the hunova^®^ robotic platform [17]. In line with the predictive model developed by Cella et al. [24], ‘fall risk’ within this study is defined as the probability of experiencing at least one fall within a 12-month follow-up period following the initial assessment. While our study protocol includes assessments at T0, T1, and T6, the Silver Index algorithm is specifically validated to provide this 12-month prospective risk horizon. The Silver Index synthesizes multiple biomechanical and clinical parameters to quantify fall risk [17,19,24,25,26,27]. These include clinical parameters, such as age, the number of recurrent falls and walking speed, and biomechanical data recorded by hunova^®^ platform, including static, dynamic and reactive balance, sensory integration, limits of stability, sit-to-stand, and gait speed. By analyzing the individual functional domains, clinicians were able to delineate the specific physical improvements (that contributed to the observed reduction in the overall risk profile. The Silver Index returns a percentage value ranging from 0% to 100%, allowing for the identification of four distinct risk categories: low (0–25%), low-medium (26–50%), medium-high (51–75%) and high risk (76–100%). Although the number of falls that occurred in the previous six months was collected at T0 and T6, these data were obtained through retrospective self-reports by patients and were therefore considered a secondary clinical outcome. The Silver Index was selected as the primary endpoint because it is based on standardized biomechanical and clinical parameters objectively acquired by the robotic platform and is not influenced by recall bias, which can affect retrospective reporting of falls [28,29,30].

Following the initial assessment and the technological evaluation of balance, the subgroup of patients undergoing rehabilitation treatment with the hunova^®^ robotic platform completed 10 sessions, held two or three times a week, each lasting 30 min. At the end of the treatment period (6 weeks, T1), the treated patients underwent a new follow-up examination and an assessment of balance and fall risk using the Silver Index. Subsequently, 6 months after the initial visit, the patients underwent a new geriatric follow-up examination and an assessment of balance and fall risk. Patients who did not undergo rehabilitation treatment also underwent a follow-up examination and an assessment of fall risk at T1 and T6.

### Statistical Analysis

As this was a retrospective study, all patients who met the study’s inclusion criteria were included, without the need for a formal sample size calculation.

The sample was described in terms of its clinical and demographic characteristics. Quantitative variables were summarized using the mean and standard deviation (SD). The Shapiro–Wilk test was used to assess the normality of the distributions.

Depending on whether or not they received rehabilitation treatment, patients were divided into two groups: the treatment group (TREAT-G), comprising those patients who underwent ten sessions of technological balance treatment, and the non-treatment group (NOTREAT-G), comprising those patients who only underwent fall risk assessment using the hunova^®^ robotic platform. Given the balanced, longitudinal design with three fixed assessment points (T0, T1, T6) and the absence of hierarchical clustering or missing data patterns, a repeated measures analysis of variance (RM-ANOVA) was selected as the primary inferential framework. This approach directly tests the within-subject time effect and the time × group interaction, which are the primary aims of this study. The choice of RM-ANOVA over more complex mixed-effects models was intended to maintain model parsimony and avoid risks of overfitting or model instability associated with complex covariance structures in a retrospective cohort. A repeated measures analysis of variance (RM-ANOVA) was then performed on the Silver Index recorded at T0, T1, and T6, considering the TREAT-G and NOTREAT-G. The comparison between groups was carried out using a two-way RM-ANOVA. Similarly, an analysis of the various functional domains (static, dynamic and reactive balance, sensory integration, stability limits, sit-to-stand, and walking speed) was performed at the three timepoints using RM-ANOVA. Comparisons between groups across the various functional areas at the three timepoints were performed using a two-way repeated measures ANOVA. Bonferroni post hoc tests were applied to all significant analyses. The level of statistical significance for each test was set at 0.05.

Secondly, the sample was divided into ‘Fallers’ (patients who reported at least one fall in the 6 months prior to the T0 assessment) and ‘No-Fallers’. For each of these two groups, the treatment group and the non-treatment group were considered. An intragroup analysis was performed using repeated-measures analysis of variance (RM-ANOVA) of the Silver Index recorded at T0, T1, and T6, considering the treatment and non-treatment Fallers groups and the treatment and non-treatment No-Fallers groups. The comparison between groups (treatment vs. non-treatment) in the Fallers and No-Fallers was performed using a two-way RM-ANOVA. Bonferroni post hoc tests were applied to all significant analyses. Statistical significance for each test was set at 0.05. Statistical analysis was performed using SPSS 25.0 (IBM Corp., Armonk, NY, USA).

## 3. Results

The study included 355 patients, of whom 54.1% (192 patients) were female, whilst the remaining 45.9% (163 patients) were male (Table 1).

Of the patients included in the study, 162 underwent rehabilitation treatment using the hunova^®^ robotic platform (TREAT-G), whilst the remaining 193 patients underwent only periodic assessments (NOTREAT-G). The two groups (TREAT-G and NOTREAT-G) showed no differences in clinical and demographic characteristics, in functional domain, or in fall risk at baseline (*p* > 0.05) (Table S1). Table 2 presents the results of the within-group analysis regarding the Silver Index, considering TREAT-G and NOTREAT-G.

**Figure 1:**
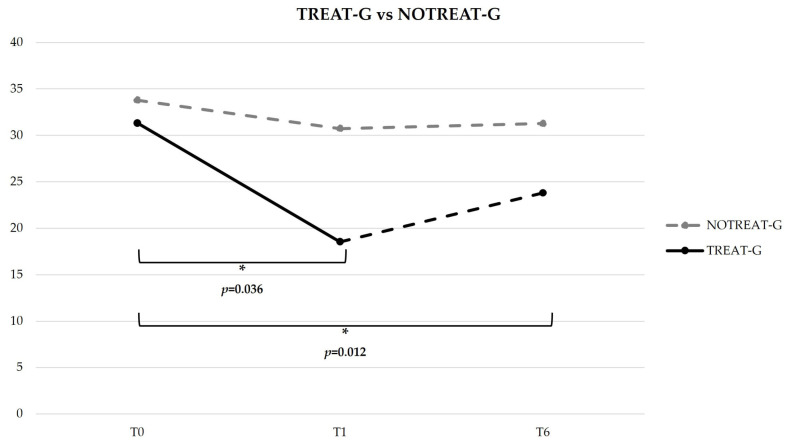
Comparison between TREAT-G and NOTREAT-G at three different timepoints. The TREAT-G trend is shown in black, while the NOTREAT-G trend is shown in gray. The solid line represents the technological treatment that was performed, while the dashed line indicates that no technological treatment was performed.

Figure 1, on the other hand, shows the comparison of the Silver Index at the three assessment points between the two groups. Figure 1 visualizes this divergence, highlighting the significant reduction in fall risk scores maintained by the TREAT-G at the 6-month follow-up compared to the relative stability or slow increase observed in the NOTREAT-G.

To identify which domains of balance have undergone a significant change, the indices of the macro-areas that make up the Silver Index were therefore analyzed: static balance, dynamic balance, reactive balance, sensory integration, limits of stability, sit-to-stand, and gait speed. Each index summarizes all the variables considered in the assessment, and a higher value indicates better performance by the subject. Table 3 shows the within-group analysis of the macro-areas that make up the Silver Index for the TREAT-G and the NOTREAT-G.

The post hoc analysis in TREAT-G revealed statistically significant results for limits of stability between T0 and T1 (*p* < 0.001), for the sit-to-stand test between T0 and T1 (*p* < 0.001) and between T0 and T6 (*p* < 0.001), and for gait speed between T0 and T1 (*p* = 0.015) and between T0 and T6 (*p* < 0.001). In NOTREAT-G, however, the post hoc analysis of indices for the various functional areas did not show statistically significant results.

As regards the comparison between the groups (Table 4), the time × group interaction showed a statistically significant difference at the three timepoints only for limits of stability (*p* = 0.004) and sit-to-stand (*p* < 0.001). After applying the Bonferroni correction, a statistically significant difference emerged between the two groups for stability limits between T0 and T6 (*p* = 0.015) and for the sit-to-stand test between T0 and T1 (*p* = 0.016) and between T1 and T6 (*p* = 0.007).

To evaluate whether the clinical and technological response to robotic intervention was influenced by a historical predisposition to instability, a secondary stratification was executed based on the participants’ fall history during the six months prior to baseline (T0). Consequently, the overall population was dichotomized into ‘Fallers’ (n = 110; 31.0%), defined as patients reporting at least one accidental fall in the preceding semester, and ‘No-Fallers’ (n = 245; 69.0%), who reported no falling events during the same timeframe. The specific clinical, demographic, and instrumental parameters of these two sub-cohorts are comprehensively presented in Table 5. Among the ‘Fallers’, 47 underwent rehabilitation treatment between T0 and T1, and 63 did not; among the ‘No-Fallers’, however, 115 underwent rehabilitation treatment between T0 and T1, and 130 did not.

Table 6 presents the RM-ANOVA results for the ‘Fallers’ and ‘No-Fallers’ (treatment group and non-treatment group) at the three timepoints. TREAT-G showed a statistically significant change over time in ‘Fallers’ (*p* = 0.016) and ‘No-Fallers’ (*p* = 0.034). In ‘Fallers’, the mean score dropped from 52.30 (T0) to 26.60 at T1, and the post hoc analysis confirmed this was a significant improvement (*p* = 0.033); this effect was sustained through T6 (21.30). The difference between T0 and T6 was highly significant (*p* = 0.010), and there was no significant change between T1 and T6. Conversely, NOTREAT-G showed no significant change over time. In ‘Fallers’, the means stayed roughly the same or even slightly increased by T6. The result was also non-significant in ‘No-Fallers’ (*p* = 0.056), though it trended toward an increase in value by T6 (39.50).

To better contextualize the long-term clinical risks associated with therapeutic absence, a cross-sectional comparison was executed between the initial, unmitigated fall risk baseline of the historical ‘Fallers’ cohort (N = 110) and the long-term observational outcome (T6) of the untreated ‘No-Fallers’ cohort (NOTREAT-G = 130).

**Figure 2:**
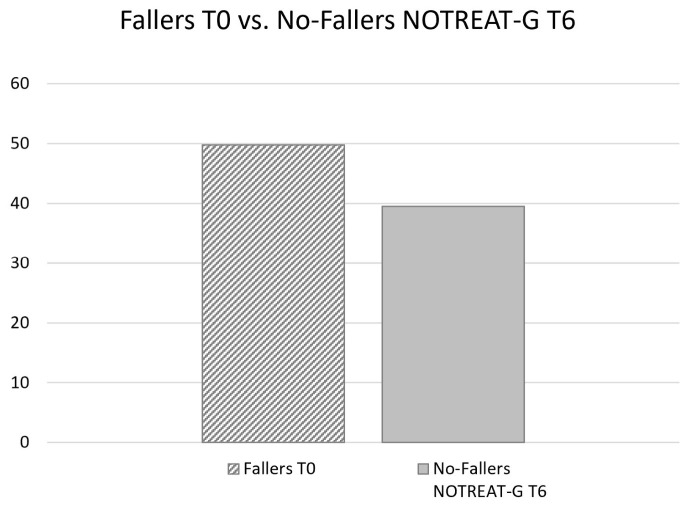
A comparison between the baseline of all ‘Fallers’ versus the long-term outcome for the ‘No-Fallers’ NOTREAT-G.

Figure 2 shows the comparison between the baseline of all ‘Fallers’ versus the long-term outcome for the ‘No-Fallers’ NOTREAT-G. It is worth noting that the ‘No-Fallers’ (NOTREAT-G) increased from 22.10 to 39.50 by T6.

The difference is narrowing even though it is still less than the ‘Fallers’ at baseline showing a “drift effect”. The ‘Fallers’ at T0 represent a group already in an advanced clinical impairment profile. While the untreated ‘No-Fallers’ group did not reach statistical significance regarding their increase in fall risk over time (*p* = 0.056), the observed upward trend suggests a potential for functional decline. This descriptive pattern highlights a possible ‘drift’ toward higher risk thresholds in the absence of intervention.

Table 7 shows the results of the comparison between the TREAT-G and NOTREAT-G groups in the ‘Fallers’ and ‘No-Fallers’.

**Figure 3:**
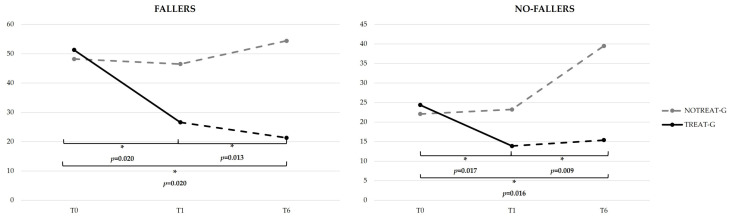
Comparison between TREAT-G and NOTREAT-G at three different timepoints in Fallers and No-Fallers. The TREAT-G trend is shown in black, while the NOTREAT-G trend is shown in gray. The solid line represents the technological treatment that was performed, while the dashed line indicates that no technological treatment was performed.

Figure 3 illustrates the results of the comparison between the ‘Fallers’ and ‘No-Fallers’. The inferential models yielded highly significant time × group interaction effects for both clinical strata, confirming divergent longitudinal pathways in the Fallers cohort (*p* = 0.006) as well as in the No-Fallers cohort (*p* = 0.029). Figure 3 contrasts the longitudinal trajectories of the Fallers and No-Fallers cohorts, providing a granular view of how robotic intervention mitigates instability across different historical fall risk profiles.

Within the ‘Fallers’, the behavioral pathways of the two treatment groups diverged significantly across every single longitudinal interval analyzed. Specifically, the TREAT-G group achieved an acutely sharper reduction in fall risk metrics during the active training phase compared to their untreated counterparts (T0–T1, *p* = 0.010). During the subsequent follow-up phase, the trajectories diverged further (T1–T6, *p* = 0.013), captured by a sustained low-risk stabilization in treated individuals contrasting with the functional degradation in controls. This cumulative effect resulted in a massive overall divergence across the complete study timeline (T0–T6, *p* = 0.015).

Within the ‘No-Fallers’, a matching, highly protective divergence pattern was identified. While initially sharing identical low-risk baseline characteristics, the change vectors between the groups diverged immediately post-treatment (T0–T1, *p* = 0.011). While the untreated ‘No-Fallers’ group did not reach statistical significance regarding their increase in fall risk over time (*p* = 0.056), the observed upward trend suggests a potential for functional decline. This descriptive pattern highlights a possible ‘drift’ toward higher risk thresholds in the absence of intervention. In addition, an intra-group analysis was carried out of the indices relating to the macro-areas that make up the Silver Index for the TREAT-G and NOTREAT-G groups, considering the ‘Fallers’ and ‘No-Fallers’ (Table S2). Only in the TREAT-G group did the post hoc analysis reveal a statistically significant difference: in the ‘Fallers’, it regarded limits of stability between T0 and T1 (*p* = 0.016) and the sit-to-stand between T0 and T1 (*p* = 0.016) and between T0 and T6 (*p* = 0.002); in the ‘No-Fallers’, however, the post hoc analysis revealed a statistically significant difference regarding stability limits between T0 and T1 (*p* = 0.015), the sit-to-stand between T0 and T1 (*p* = 0.001) and between T0 and T6 (*p* = 0.002), and gait speed between T0 and T6 (*p* < 0.001).

As for the comparison between the groups—specifically in the ‘Fallers’ and the ‘No-Fallers’ between TREAT-G and NOTREAT-G—the time × group interaction revealed a statistically significant difference at the three assessment timepoints only for stability limits (*p* = 0.036), sit-to-stand test (*p* = 0.050), and gait speed (*p* = 0.029) for ‘Fallers’ and only for limits of stability (*p* = 0.025), the sit-to-stand test (*p* = 0.034), and gait speed (*p* = 0.018) for ‘No-Fallers’ (Table S3). After applying the Bonferroni correction, a statistically significant difference emerged between the two groups in ‘Fallers’ for limits of stability between T0 and T6 (*p* = 0.016), for the sit-to-stand test between T1 and T6 (*p* = 0.010), and for gait speed between T1 and T6 (*p* = 0.013) and between T0 and T6 (*p* = 0.010). Similarly, following the application of post hoc tests among the ‘No-Fallers’, a statistically significant difference emerged between the two groups—TREAT-G vs. NOTREAT-G—regarding limits of stability between T0 and T6 (*p* = 0.014), for the sit-to-stand test between T1 and T6 (*p* = 0.016), and for gait speed between T0 and T6 (*p* = 0.012).

## 4. Discussion

The field of neurological and geriatric rehabilitation is changing towards using objective, sensor-driven techniques for assessing risk and creating tailored treatments. This paradigm shift has been facilitated by continuous advances in sensing technologies, signal processing, and precision measurement systems. Recent engineering developments have demonstrated that accurate signal phase adjustment and displacement estimation are essential for improving the reliability and robustness of sensor-based electromechanical systems used in motion monitoring and control applications [31]. In this context, robotic platforms provide an opportunity to obtain objective and reproducible measures of motor performance and fall risk, reducing the limitations associated with subjective clinical assessments. The findings of this retrospective, observational study show that a structured, robot-assisted training program using the hunova^®^ robotic platform leads to significant and lasting reductions in fall risk, as measured by the validated multi-factorial Silver Index algorithm. It is important to note that the Silver Index acts as a composite metric of fall risk. While it incorporates both clinical and biomechanical inputs, the observed reduction in the total score is statistically underpinned by significant improvements in specific functional sub-domains—specifically gait speed and limits of stability—which serve as objective indicators of physical performance following robotic rehabilitation.

The study sample is characterized by marked heterogeneity, both in terms of age and baseline clinical condition; whilst this represents a limitation in terms of homogeneity, it is consistent with the observational nature of the study. The sample examined is also characterized by a fall risk falling within the medium-high category; nevertheless, approximately two-thirds of the assessed population reported never having fallen in the 6 months prior to the assessment. Furthermore, considering the entire sample, it can be seen that approximately 45% underwent robotic rehabilitation treatment to improve balance and reduce the risk of falling using the hunova^®^ robotic platform: data analysis showed that the group of patients who underwent rehabilitation treatment experienced a significant reduction in the risk of falling. The significant reduction in fall risk was observed immediately after training, and notably, the improvement was maintained significantly even at the assessment carried out 6 months after baseline, confirming the stability of the gains achieved through robotic rehabilitation using the hunova robotic platform. In the group of patients who did not undergo the treatment, however, the risk of falling, as assessed using the Silver Index, remained essentially unchanged. These results suggest that the use of personalized and adaptive protocols delivered via hunova^®^, specifically designed to improve balance and reduce the risk of falling, enables a rapid and significant reduction in the risk of falling, which tends to remain stable over time.

As described above, the Silver Index is a composite score which, in addition to taking clinical factors into account, also incorporates a multifactorial profile of fall risk developed by Cella and colleagues [24]. The areas examined that contribute to the definition of fall risk are static, dynamic, and reactive balance; sensory integration; limits of stability; the transition from a seated to a standing position; and gait speed. A more detailed analysis of the individual functional domains of the Silver Index revealed, in the treated patients, statistically significant improvements in the areas relating to limits of stability, the ability to rise from a seated position, and gait speed. The ability to rise from a seated position and limits of stability improved statistically significantly when comparing the two groups, treated and untreated. Some authors have highlighted how impaired physical functions, such as reduced walking speed, increase the risk of falling [32,33]. It is interesting to note that the indices relating to dynamic and reactive balance, which are also fundamental for fall prevention [34], show an improvement that does not, however, reach statistical significance. All the macro-areas showing a significant improvement or those bordering on significance are of a dynamic nature: this underscores how dynamic balance is an important indicator of mobility [35]. This highlights how technology-assisted balance training enables a significant improvement in dynamic postural functions, which are fundamental for fall prevention. An in-depth analysis of specific functional domains confirmed that the improvements were driven by significant, sustained changes in limits of stability, sit-to-stand, and gait speed (Table S3). These interaction effects underscore the robust and multidimensional impact of the intervention across different clinical risk profiles.

As described above, around two-thirds of the sample surveyed reported that they had not fallen at all in the six months prior to the assessment. Compared with the ‘Fallers’, the ‘No-Fallers’ had a significantly lower average age and a significantly lower risk of falling. The analysis of the ‘Fallers’ and ‘No-Fallers’ subgroups revealed a statistically significant improvement in the Silver Index in the period immediately following treatment and six months after the baseline assessment; the ‘Fallers’ who did not undergo treatment, however, showed no statistically significant changes in the Silver Index at the three timepoints. The ‘No-Fallers’ who underwent treatment reported a statistically significant improvement in the Silver Index immediately after training and six months after the baseline assessment; it is interesting to note, however, that the ‘No-Fallers’ who did not undergo treatment showed a worsening trend in fall risk over the six-month period, although this did not reach statistical significance. These results highlight how the risk of falling is effectively reduced in adult and elderly patients who report not falling and who have a low risk of falling and that this risk may increase over time in individuals who are potentially at risk who do not follow a tailored rehabilitation program, although this trend did not reach clear statistical significance in the present study.

It is also interesting to note that the six-month risk of falls of ‘No-Fallers’ who did not undergo treatment changes to the point of nearly reaching the baseline value of Fallers: in other words, over a six-month period, the risk of falling of patients who report not having fallen—who start with a low risk of fall—changes almost as much as the risk of fall of patients who report falling at baseline. It is noteworthy that untreated ‘No-Fallers’ showed an increase in Silver Index values over the 6-month follow-up period. Although this change did not reach statistical significance (*p* = 0.056), it suggests a possible tendency toward worsening balance performance and fall risk profile in the absence of a targeted rehabilitation intervention. However, given the lack of statistical significance, this finding should be interpreted with caution and considered hypothesis-generating rather than conclusive. Furthermore, comparisons between ‘Fallers’ at baseline and untreated ‘No-Fallers’ at follow-up were performed for descriptive purposes only and should not be interpreted as evidence of longitudinal progression or causal transition between risk categories. The divergence between the treated and untreated groups was sustained through the six-month follow-up. The absence of treatment allows for a deterioration in ‘No-Fallers’ to levels that mimic the high-risk baseline, emphasizing the necessity of intervention even in currently stable populations. This confirms that while the untreated trended toward the higher-risk baseline values of the ‘Fallers’, the TREAT-G maintained a significantly lower and safer profile, effectively widening the statistical gap between treated and untreated individuals over time. Future prospective studies with longer follow-up periods are warranted to determine whether these observed trends correspond to clinically meaningful changes in fall risk over time.

Although the untreated ‘No-Fallers’ showed a numerical increase in Silver Index scores over time, the lack of clear statistical significance prevents us from drawing definitive conclusions regarding the natural progression of fall risk in this subgroup. However, untreated low-risk individuals developed a risk profile extremely similar to that of high-risk individuals at baseline for the importance of early intervention. This trend is of particular interest to clinicians, as it suggests that even non-fallers with a low risk of falling can be included in a rehabilitation program to prevent falls by improving or strengthening postural strategies.

Although randomized controlled trials (RCTs) serve as the gold standard for evaluating the effectiveness of rehabilitation interventions, it is well established that data from real-world clinical settings provide a crucial additional insight into the generalizability and applicability of the results. In this context, while recent controlled studies [36] have analyzed relatively homogeneous populations and highly standardized protocols, the present study reflects a more heterogeneous clinical population, comprising subjects with neurological, orthopedic, and vestibular conditions across a wide age range. This heterogeneity strengthens the external validity of the results and supports the implementation of robot-assisted interventions in daily clinical practice. In recent years, a growing number of studies have demonstrated that robotic therapies can improve functional outcomes and balance. Innovative research, such as that conducted by Ozaki et al. [37], has shown significant improvements in lower limb strength and dynamic balance in frail older adults. More recent randomized studies have since demonstrated the safety and efficacy of robotic balance training systems, resulting in improvements in mobility and functional independence comparable to those of traditional rehabilitation [38]. Systematic reviews and meta-analyses show consistent gains in walking speed, balance, and functional abilities across a range of populations—including those with neurological conditions—further corroborate these findings [39,40]. These results lend credence to the theory that robotic therapies, which are essential components of functional recovery, promote motor learning through high-intensity, repetitive, and task-specific training. Accordingly, the results of the present study are consistent with those of recent randomized trials. In particular, the multicenter study by Kwag et al. [36] demonstrated that a combined intervention (home exercises and robot-assisted training) significantly reduces the risk of falls, as determined by the Silver Index. The intervention group recorded a lower percentage of participants who experienced falls, although there was no statistically significant reduction in fall risk. The in-depth examination of specific factors influencing fall risk is a unique aspect of the retrospective study presented here. This approach allows for a more detailed description of motor function, including static, dynamic, and reactive balance; walking speed; and the ability to rise from a seated position, unlike many RCTs that focus on composite indices or fall incidence. This analysis highlights the fundamental importance of improved postural control and greater functional mobility and offers significant insights into how robot-assisted therapies can reduce the risk of falling. In fact, previous research has demonstrated a strong correlation between a reduced risk of falling and improvements in certain aspects of balance, such as stability limits and gait flexibility [41]. From a clinical perspective, the findings support the use of robotic platforms, such as hunova^®^, in rehabilitation programs for fall prevention. These devices allow for the precise measurement of balance disorders and the delivery of personalized and adaptive therapies, which can improve motor learning and treatment adherence. According to recent systematic reviews, these technologies have the potential to improve functional outcomes in neurological and geriatric populations [42]. Additionally, data from ordinary clinical practice, including procedures that were shorter and more sustainable than those usually employed in RCTs, could be analyzed thanks to the retrospective design. In line with earlier observational and interventional studies, the findings imply that even comparatively little exposure to robot-assisted training can yield quantifiable gains on parameters related to fall risk [43].

While these findings show the clinical efficacy of hunova^®^, it is important to acknowledge the current financial barriers to implementation. This technology is currently positioned as a high-tier clinical tool. However, the long-term social and economic impact of fall prevention—reducing the need for acute hospitalizations, surgical interventions, and subsequent nursing care—may offset these initial costs over time, especially considering direct and indirect social costs for patients and caregivers. As with many emerging medical technologies, costs will likely normalize with broader clinical adoption and market maturation. Future studies should focus on formal cost-effectiveness analyses to determine the ‘break-even’ point for clinical facilities implementing these devices.

Despite the interesting nature of these findings, some limitations in the study should be recognized. The retrospective and observational design of this study does not have the strict environmental control and true randomization found in clinical trials. This introduces possible conflicting variables due to the diverse clinical causes and etiologies among the participants. Also, tracking fall risk metrics over six months is useful but does not measure very long-term functional retention or overall long-term fall rates. Future research should use these real-world data points to create large-scale, prospective randomized controlled trials focused on evaluating early robotic prevention strategies in asymptomatic, at-risk populations. Another potential limitation of this study is that the actual incidence of falls was not selected as the primary endpoint. Although falls represent the most clinically significant outcome, their assessment in this retrospective study was based on patients’ recall over a six-month period. Previous research has shown that retrospective reporting of falls may underestimate or overestimate the actual number of falls compared to prospective monitoring via calendars or regular follow-up [28,29,30]. In contrast, the Silver Index provides an objective and standardized assessment of fall risk based on instrumentally recorded biomechanical and clinical variables. Furthermore, the algorithm underlying the Silver Index has been prospectively validated against future falls in older adults, demonstrating good predictive performance [24]. For these reasons, we considered the Silver Index to be the most robust outcome available in the current set of retrospective real-world datasets. However, future prospective studies should combine objective measures of fall risk with prospectively recorded fall incidence to further validate the long-term clinical impact of robotic rehabilitation. Furthermore, while no statistically significant differences were observed between treatment groups at baseline—suggesting that the cohorts were well-matched—it is not possible to entirely rule out the influence of unmeasured residual confounding factors, a common limitation in retrospective observational designs.

## 5. Conclusions

In conclusion, the results of this study highlight how the use of personalized robot-assisted rehabilitation protocols, delivered via the hunova^®^ robotic platform, leads to significant and sustained reductions in fall risk across a heterogeneous clinical population. This improvement is already evident at the end of the intervention and remains stable six months later, suggesting an effect that is consolidated over time. The analysis of functional domains has also shown that the main benefits relate to dynamic aspects of postural function, such as stability limits, the ability to perform sit-to-stand, and gait speed, confirming the central role of the dynamic component in balance control.

The results obtained in the ‘Fallers’ and ‘No-Fallers’ subgroups highlight that the intervention is effective from both a rehabilitative and a preventive perspective. In particular, the absence of treatment in low-risk individuals appears associated with a progressive worsening in the risk of falls over time—a descriptive finding that, while requiring further investigation in prospective studies, underscores a potential critical therapeutic window for early, targeted preventative interventions.

Given the pivotal role of economic viability in clinical adoption, future investigations should prioritize formal health-economic evaluations. By comparing the cost of robotic interventions against the direct and indirect savings generated by the prevention of fall-related injuries, we aim to provide healthcare stakeholders with the necessary data to justify long-term investments in these high-precision technologies.

Overall, these data support the integration of robot-assisted rehabilitation into clinical pathways for fall prevention, even in populations that appear to be at low risk in order to preserve motor function and reduce the future incidence of adverse events. By shifting healthcare paradigms towards early, proactive technological training and away from reactive post-event treatments, clinicians will be able to preserve long-term motor independence, slow natural age-related and disease-driven regression, and significantly mitigate the public health burden of accidental fall events.

## Figures and Tables

**Table 1:** Clinical and instrumental characteristics at baseline.

		N = 355
**Gender**	W vs. M	192 vs. 163
**Age**	mean ± SD	58.24 ± 19.63
**Pathology**	Geriatric	114
	Orthopedic	44
	Stroke	57
	PD	31
	MS	49
	Vestibular Impairment	22
	Visual Impairment	38
**Falls in previous 6 months**	Y vs. N	110 vs. 245
**Silver Index T0**	mean ± SD	34.25 ± 14.54
**Robotic Treatment**	Y vs. N	162 vs. 193

W: Women; M: Man; PD: Parkinson’ Disease; MS: Multiple Sclerosis.

**Table 2:** Repeated measures ANOVA and post hoc analysis for Silver Index in the TREAT-G and NOTREAT-G.

	T0 Mean ± SD	T1 Mean ± SD	T6 Mean ± SD	*p* Value	T0–T1	T1–T6	T0–T6
**TREAT-G**	31.34 ± 22.33	18.55 ± 19.47	23.83 ± 17.21	**0.036**	**<0.001**	0.519	**0.009**
**NOTREAT-G**	33.79 ± 23.42	30.73 ± 19.18	31.28 ± 16.66	0.309	-	-	-

The significant values for *p* < 0.005 are in bold. The significant values from the post hoc test (*p* < 0.016) are in bold.

**Table 3:** The repeated measures ANOVA for the components of the Silver Index in the TREAT-G and NOTREAT-G.

	TREAT-G				NOTREAT-G			
	T0 Mean ± SD	T1 Mean ± SD	T6 Mean ± SD	*p* Value	T0 Mean ± SD	T1 Mean ± SD	T6 Mean ± SD	*p* Value
**Static Balance**	−0.46 ± 1.26	−0.16 ± 1.01	−0.37 ± 1.49	0.300	−0.34 ± 1.25	−0.33 ± 1.20	−0.26 ± 0.84	0.242
**Dynamic Balance**	0.18 ± 0.85	0.36 ± 2.35	0.56 ± 0.87	0.054	0.18 ± 0.92	0.64 ± 0.82	0.68 ± 0.78	0.078
**Reactive Balance**	0.21 ± 1.60	0.62 ± 0.77	0.65 ± 0.70	0.053	0.28 ± 1.00	0.75 ± 0.53	0.77 ± 0.59	0.179
**Sensory Integration**	−0.91 ± 1.88	−0.52 ± 1.42	−0.61 ± 1.77	0.221	−0.59 ± 2.85	−0.34 ± 0.98	−0.59 ± 1.31	0.618
**Limits of Stability**	0.327 ± 0.70	1.03 ± 5.41	0.55 ± 0.40	**<0.001**	0.51 ± 2.31	0.74 ± 0.53	0.60 ± 0.45	0.146
**Sit-to-Stand**	−2.29 ± 2.72	−1.53 ± 2.27	−0.81 ± 1.79	**<0.001**	−1.55 ± 2.31	0.03 ± 1.09	0.05 ± 0.89	0.101
**Gait Speed**	−1.18 ± 1.02	−0.82 ± 1.09	−0.46 ± 0.93	**<0.001**	−0.77 ± 1.14	−0.36 ± 0.86	0.45 ± 4.77	0.123

The significant values for *p* < 0.005 are in bold.

**Table 4:** Two-way repeated measures ANOVA and post hoc analysis for the components of the Silver Index.

TREAT-G vs. NOTREAT-G					
			Post Hoc Test		
	*p* Time	*p* Time × Group	T0–T1	T1–T6	T0–T6
**Static Balance**	0.927	0.248	-	-	-
**Dynamic Balance**	0.269	0.415	-	-	-
**Reactive Balance**	0.033	0.409	-	-	-
**Sensory Integration**	0.173	0.460	-	-	-
**Limits of Stability**	0.011	**0.004**	0.156	0.693	**0.015**
**Sit-to-Stand**	<0.001	**<0.001**	**0.016**	**0.007**	0.125
**Gait Speed**	0.003	0.054	-	-	-

The significant values for *p* < 0.005 are in bold. The significant values from the post hoc test (*p* < 0.016) are in bold.

**Table 5:** Clinical and instrumental characteristics at baseline in ‘Fallers’ and ‘No-Fallers’.

		Fallers (n = 110)	No-Fallers (n = 245)
**Gender**	W vs. M	69 vs. 41	123 vs. 122
**Age**	mean ± SD	67.8 ± 18.4	53.9 ± 18.6
**Pathology**	Geriatric	52	62
	Orthopedic	7	37
	Stroke	13	44
	PD	4	27
	MS	17	32
	Vestibular Impairment	12	10
	Visual Impairment	5	33
**Falls in previous 6 months**	None	0	245
	1 fall	45	0
	≥2 falls	65	0
**Silver Index T0**	mean ± SD	49.75 ± 13.44	23.25 ± 17.19
**Robotic treatment**	Y vs. N	47 vs. 63	115 vs. 130

W: Women; M: Man; PD: Parkinson’s Disease; MS: Multiple Sclerosis.

**Table 6:** Repeated measures ANOVA and post hoc analysis for Silver Index in ‘Fallers’ and ‘No-Fallers’ for TREAT-G and NOTREAT-G.

Fallers							
	T0 Mean ± SD	T1 Mean ± SD	T6 Mean ± SD	*p* Value	T0–T1	T1–T6	T0–T6
**TREAT-G**	52.30 ± 23.39	26.60 ± 17.42	21.30 ± 21.12	**0.016**	**0.033**	0.987	**0.010**
**NOTREAT-G**	48.20 ± 22.82	46.50 ± 7.94	54.40 ± 8.63	0.981	-	-	-
**No-Fallers**							
	**T0** **Mean ± SD**	**T1** **Mean ± SD**	**T6** **Mean ± SD**	***p*** **Value**	**T0–T1**	**T1–T6**	**T0–T6**
**TREAT-G**	24.40 ± 20.20	13.90 ± 20.30	21.40 ± 15.20	**0.034**	**0.046**	0.054	**0.040**
**NOTREAT-G**	22.10 ± 15.90	23.23 ± 13.0	39.50 ± 17.80	0.056	-	-	-

The significant values for *p* < 0.005 are in bold. The significant values from the post hoc test (*p* < 0.016) are in bold.

**Table 7:** Comparison between TREAT-G and NOTREAT-G in Fallers and No-Fallers.

TREAT-G vs. NOTREAT-G					
	*p* Time	*p* Time × Group	T0–T1	T1–T6	T0–T6
**Fallers**	**<0.001**	**0.006**	**0.010**	**0.013**	**0.015**
**No-Fallers**	**0.012**	**0.029**	**0.011**	**0.015**	**0.016**

The significant values for *p* < 0.005 are in bold. The significant values from the post hoc test (*p* < 0.016) are in bold.

## Data Availability

The data presented in this study are available on request from the corresponding author due to privacy and ethical restriction.

## Supplementary Materials

The following supporting information can be downloaded at https://www.mdpi.com/article/10.3390/s26134088/s1, Table S1: Baseline clinical and instrumental characteristics of TREAT-G and NOTREAT-G. Table S2: The RM-ANOVA for the components of the Silver Index in TREAT-G and NOTREAT-G, considering ‘Fallers’ and ‘No-Fallers’. Table S3: Comparison for the components of the Silver Index between TREAT-G and NOTREAT-G in ‘Fallers’ and ‘No-Fallers’.
