# Supplementary material for: The Effectiveness of Personalized Robot-Assisted Rehabilitation on Fall Risk: A Retrospective Controlled Study with a 6-Month Follow-Up
Source: Sensors (Basel). 2026 Jun 27;26(13):4088. doi: 10.3390/s26134088 (PMC13363911; doi:10.3390/s26134088)
Supplement: Supplementary file 1 [file sensors-26-04088-s001.zip › sensors-4364029-supplementary.pdf]

Table S1. Baseline clinical and instrumental characteristics of TREAT-G and NOTREAT-G.

		TREAT-G n=162	NOTREAT-G n=193
**Gender**	W vs M	81 vs 81	111 vs 82
**Age**	mean ± SD	59.21 ± 17.06	58.69 ± 21.30
**Pathology**	Geriatric	39	75
	Orthopedic	7	37
	Stroke	37	20
	PD	18	13
	MS	29	20
	Vestibular Impairment	15	7
	Visual Impairment	17	21
**Falls in previous 6 months**	Y vs N	47 vs 114	63 vs 131
**Silver Index T0**	mean ± SD	31.34 ± 22.33	33.78 ± 23.42

W: Women; M: Man; PD: Parkinson' Disease; MS: Multiple Sclerosis

Table S2. RM-ANOVA for the components of the Silver Index in the TREAT-G and NOTREAT-G, considering 'Fallers' and 'No-Fallers'.

	TREAT-G				NOTREAT-G			
	T0 mean ± SD	T1 mean ± SD	T6 mean ± SD	p value	T0 mean ± SD	T1 mean ± SD	T6 mean ± SD	p value
**FALLERS**								
Static Balance	-1.14 ± 1.65	-0.39 ± 0.92	-0.84 ± 1.38	0.151	-0.63 ± 1.21	0.06 ± 0.63	-0.21 ± 0.78	0.230
Dynamic Balance	0.03 ± 1.03	0.02 ± 0.75	0.06 ± 1.32	0.247	-0.04 ± 0.91	1.01 ± 0.50	1.10 ± 0.23	0.172
Reactive Balance	-0.15 ± 1.26	0.52 ± 0.70	0.51 ±0.60	**0.016**	0.63 ± 1.23	0.67 ± 0.63	0.75 ± 0.55	0.215
Sensory Integration	-1.71 ± 2.53	-0.93 ± 1.86	-0.50 ± 1.24	**0.037**	-0.71 ± 1.39	-0.44 ± 0.96	-0.63 ± 1.16	0.378
Limits of Stability	0.58 ± 0.76	0.11 ± 0.33	0.10 ± 0.37	**0.006**	0.19 ± 0.73	0.30 ± 0.77	0.38 ± 0.40	0.352
Sit to Stand	-3.42 ± 3.32	-2.13 ± 2.31	-0.98 ± 1.88	**< 0.001**	-2.33 ± 2.56	-1.79 ± 1.49	-1.49 ± 0.41	0.092
Gait Speed	-1.65 ± 0.81	-1.24 ± 0.99	-1.03 ± 0.72	**0.006**	-1.20 ± 1.03	-0.90 ± 0.78	-1.60 ± 0.71	0.167
**NO FALLERS**								
Static Balance	-0.18 ± 0.94	-0.06 ± 1.03	-0.19 ± 1.50	0.929	-0.21 ± 1.25	-0.39 ± 1.26	-0.27 ± 0.87	0.335
Dynamic Balance	0.24 ± 0.78	0.20 ± 2.79	0.34 ± 0.63	0.265	0.29 ± 0.91	0.58 ± 0.85	0.59 ± 0.83	0.348
Reactive Balance	0.46 ± 1.70	0.66 ± 0.80	0.78 ± 0.82	**0.047**	0.40 ± 0.86	0.76 ± 0.52	0.77 ± 0.61	0.198
Sensory Integration	-0.58 ± 1.43	-0.33 ± 1.12	-0.65 ± 1.95	0.990	-0.54 ± 3.32	-0.33 ± 0.99	-0.57 ± 1.36	0.433
Limits of Stability	0.43 ± 0.64	0.28 ± 6.56	0.16 ± 0.40	**0.007**	0.66 ± 0.52	0.76 ± 0.50	0.58 ± 0.47	0.190
Sit to Stand	-1.83 ± 2.29	-1.25 ± 2.20	-0.75 ± 1.78	**< 0.001**	-1.18 ± 2.09	-1.07 ± 1.02	-1.15 ± 0.96	0.422
Gait Speed	-0.99 ± 1.04	-0.62 ± 1.08	-0.25 ± 0.91	**< 0.001**	-0.57 ± 1.13	-0.27 ± 0.84	-0.67 ± 5.23	0.167

The significant values for p < 0.005 are in bold.
The significant values from the post-hoc test (p < 0.016) are in bold

Table S3. Comparison for the components of the Silver Index between TREAT-G and NOTREAT-G in 'Fallers' and 'No-Fallers'.

	TREAT-G vs NOTREAT-G				
			Post-hoc test		
	p (time)	p (time×group)	T0-T1	T1-T6	T0-T6
**FALLERS**					
Static Balance	0.330	0.370	-	-	-
Dynamic Balance	0.059	0.582	-	-	-
Reactive Balance	0.031	0.429	-	-	-
Sensory Integration	0.085	0.408	-	-	-
Limits of Stability	0.017	**0.036**	0.034	0.019	**0.016**
Sit to Stand	<0.001	**0.050**	0.043	**0.010**	0.078
Gait Speed	<0.001	**0.029**	0.072	**0.013**	**0.010**
**NO FALLERS**					
Static Balance	0.655	0.537	-	-	-
Dynamic Balance	0.360	0.331	-	-	-
Reactive Balance	0.161	0.615	-	-	-
Sensory Integration	0.400	0.422	-	-	-
Limits of Stability	0.076	**0.025**	0.208	0.547	**0.014**
Sit to Stand	<0.001	**0.034**	0.708	**0.016**	0.043
Gait Speed	0.015	**0.018**	0.931	0.776	**0.012**

The significant values from the post-hoc test ($p < 0.016$) are in bold.
